# The performance of ensemble-based free energy protocols in computing binding affinities to ROS1 kinase

**DOI:** 10.1038/s41598-022-13319-6

**Published:** 2022-06-21

**Authors:** Shunzhou Wan, Agastya P. Bhati, David W. Wright, Alexander D. Wade, Gary Tresadern, Herman van Vlijmen, Peter V. Coveney

**Affiliations:** 1 Department of Chemistry, Centre for Computational Science, University College London, London, UK; 2 Computational Chemistry, Janssen Research & Development, Turnhoutseweg 30, 2340 Beerse, Belgium; 3 Advanced Research Computing Centre, University College London, London, WC1H 0AJ UK; 4 Computational Science Laboratory, Institute for Informatics, Faculty of Science, University of Amsterdam, Amsterdam, The Netherlands

**Keywords:** Computational models, Computational biology and bioinformatics, Drug discovery, Molecular medicine

## Abstract

Optimization of binding affinities for compounds to their target protein is a primary objective in drug discovery. Herein we report on a collaborative study that evaluates a set of compounds binding to ROS1 kinase. We use ESMACS (enhanced sampling of molecular dynamics with approximation of continuum solvent) and TIES (thermodynamic integration with enhanced sampling) protocols to rank the binding free energies. The predicted binding free energies from ESMACS simulations show good correlations with experimental data for subsets of the compounds. Consistent binding free energy differences are generated for TIES and ESMACS. Although an unexplained overestimation exists, we obtain excellent statistical rankings across the set of compounds from the TIES protocol, with a Pearson correlation coefficient of 0.90 between calculated and experimental activities.

## Introduction

ROS1 is a receptor tyrosine kinase closely related to the anaplastic lymphoma kinase (ALK) and leukocyte tyrosine kinase (LTK) based on sequence similarity of their kinase domains. The ROS1 protein is composed of an extracellular domain containing several fibronectin-like repeats and a cytoplasmic kinase domain. Genomic rearrangements involving ROS1 have been detected in a variety of cancers including non-small cell lung cancer (NSCLC), glioblastoma, and colorectal cancer amongst others. Chimeric fusion proteins involving ROS1 fused to the N-terminal domains of different unrelated proteins have been shown to be oncogenic^1^. Marketed kinase inhibitor drugs such as Crizotinib and Entrecitinib are active at various kinases including ALK and ROS1 and are used in the treatment of NSCLC. Although the rate of oncogenic ROS1 fusions is generally low, for instance 1–2% in NSCLC, it may be higher in other cancers^2, 3^. Consequently, the search for ROS1-targeted therapies is the subject of extensive investigation.

The development of a new drug is an expensive and time-consuming process that takes around 10–15 years with an average cost of US $ 1.3–2.9 billion^4, 5^. Virtual screening approaches have been increasingly applied in the drug discovery process, evidenced by their involvement in more than 70 approved drugs^6^. In most cases, computational approaches have been extensively used in the initial stages including hit to lead and lead optimisation. They can offset the costs associated with bringing novel drugs to market, and accelerate the preclinical drug screening process.

Docking is the most prominently used virtual screening approach. It has been employed as a fast way to predict whether a given compound is able to bind to a specific target, and to estimate the putative bound conformation of the compound. Docking approaches perform well at recapitulating binding modes and assessing the fit of molecules into binding sites. Furthermore, this placement ability allows them to separate active and inactive molecules and provide enriched selections with greater chance of showing bioactivity. However, the docking scores are generally not an accurate indicator for the binding energies and cannot rank the activity of structurally similar molecules. Consequently, more sophisticated approaches are needed that on the one hand capture the dynamic nature of protein ligand binding, whilst also providing a more correct physical assessment of the interaction and binding energies. Molecular dynamics simulations are well suited to study the dynamics of the proteins, refined binding poses, and more importantly, generate more reliable predicted binding affinities.

In recent years, a lot of effort has been invested to develop workflows that simplify and automate the process of free energy calculations^7^, which includes steps to plan, set up and execute simulations, and to analyse the final results. A few automated pipelines have been developed for free energy calculations, including commercial ones like FEP+^8^ and Molecular Operating Environment (MOE)^9^, and non-commercial ones like Amber free energy workflow (FEW)^10^, FESetup^11^, FEPrepare^12^, CHARMM-GUI FEP calculator^13^, to name just a few. Our group also developed an automatic, high throughput workflow to run free energy simulations, known as the binding affinity calculator (BAC)^23^. The workflows have found promising applications in academia as well as industry. FEP+^8^, for example, is the free energy calculation suite from Schrödinger Inc. that has made a significant impact in the pharmaceutical industry. To obtain reliable and reproducible free energy estimation, we have developed two ensemble-based protocols in the last few years, named ESMACS (enhanced sampling of molecular dynamics with approximation of continuum solvent)^7, 14^ and TIES (thermodynamic integration with enhanced sampling)^7, 15^. ESMACS is an end-point approach based on the molecular mechanics Poisson-Boltzmann surface area method (MMPBSA)^16^, and TIES is an alchemical approach centred on thermodynamic integration (TI). Our group has recently publicly released the comprehensive TIES toolkit (https://www.ties-service.org/) to automatically setup, execute, and analyse such calculations. We recently undertook a systematic uncertainty quantification (UQ) analysis of molecular simulations and binding free energy estimations, and showed that ensembles are required to acquire robust statistical measures of uncertainty, which address both parametric and stochastic components of MD simulations^17^. The names of our methods emphasise the central importance of the ensemble-based nature of the protocols employed^7, 14, 18^. Ensemble approaches lead to increased reliability and reproducibility, with tighter control of standard errors revealing the distribution of results that can be obtained^7, 19–22^. ESMACS and TIES are performed using a binding affinity calculator (BAC)^23^, a computational pipeline designed to automate the end-to-end execution of free energy calculations, and to handle ensemble calculations.

The purpose of the present study is to evaluate the ability of ESMACS and TIES to estimate binding affinities of a set of 150 compounds to the ROS1 protein target. Like many protein kinases, the binding site of ROS1 is well structured, and the binding mode for the scaffold of the congeneric series is clearly defined in the crystal structure. Under such circumstances, it is reasonable to expect that good predictions can be achieved, although rotatable bonds in some of the compounds may pose a challenge for conformational sampling and hence convergence of the predictions.

## Methods and simulations

In this section, we first describe the dataset of the compounds and the preparation of the molecular models before explaining the two methodologies used to predict binding affinity values.

### Compound set

**Figure 1:**
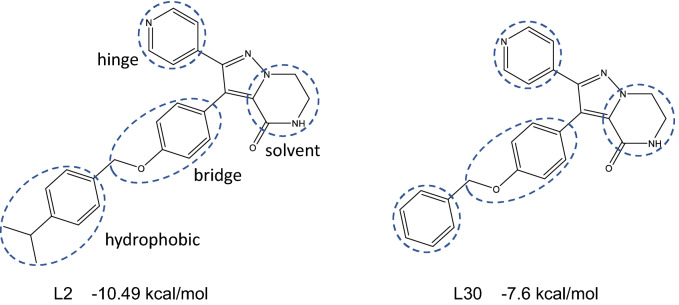
Compounds L2 and L30, with which other compounds are paired for the TIES simulations. The cycles show where the modifications are made. The four positions, at which variations are made, are labelled as: hinge (hg) which interacts with the hinge of the kinase, hydrophobic (hy) which binds in a hydrophobic pocket, solvent (s) which points toward the bulk solvent, and bridge (b) which sits in the middle of the compounds.

A set of kinase inhibitor candidates for ROS1 was available from Janssen, which comprises 150 compounds from an internal ROS1 inhibition lead optimization program. The compounds are numbered as per the order in the file provided by Janssen. They are all based on the same chemical scaffold, a tetrahydropyrazolopyrazinone (Fig. 1). The ROS1 activity is provided from a biochemical substrate inhibition assay and a cell proliferation assay. The former is preferred here for comparing with calculated binding free energies. The pIC_50_ of the compounds ranged from 5.03 to 8.37, a total of 3.34 log units (no activity was detected for five of the compounds at the highest concentration (10 μM) tested; a pIC_50_ of 5.00 was used hereafter). To capture the experimental variability, we extracted all data for repeat bioactivity measurements of the same compound in the same assay. The average standard deviation in pIC_50_ for compounds tested at least twice in the ROS1 biochemical assay was 0.12 log units. This is considered an underestimate of the true experimental error which should also include considerations of new compound samples, different assay conditions and laboratories etc.

The compounds differ with functional groups added at different positions of the scaffold (Fig. 1), which interact with different regions of the binding pocket^24^. The hinge (hg) position interacts with the adenine region of the pocket, where one hydrogen bond forms with a hinge residue. The functional groups at the hydrophobic (hy) position binds in a hydrophobic pocket. The solvent (s) position interacts with a solvent exposure region, and also partial exposes to the bulk solvent. The atoms at bridge (b) position sit in the middle of the compounds, and interact with a buried region at the bottom of the binding pocket. Modifications at these positions can change the binding affinity and/or the selectivity of the compounds. The compounds can be grouped based on their electrostatic properties (charged and non-charged), or the positions where functional groups are added. The DFG kinase motif is a conserved Asp-Phe-Gly tripeptide at the proximal end of a flexible activation loop (A-loop). Two distinct conformations, DFG-in and DFG-out, are often observed in the kinase structures, which indicate the catalytic states of the kinases. There are two ROS1 X-ray structures in the protein data bank (PDB IDs: 4UXL and 3ZBF), both in an active DFG-in conformation. Janssen have obtained four crystallographic structures. All of them have an inactive DFG-out conformation and are complexed with compounds that share the same scaffold (Fig. 1).

### Model preparation and molecular simulation

**Figure 2:**
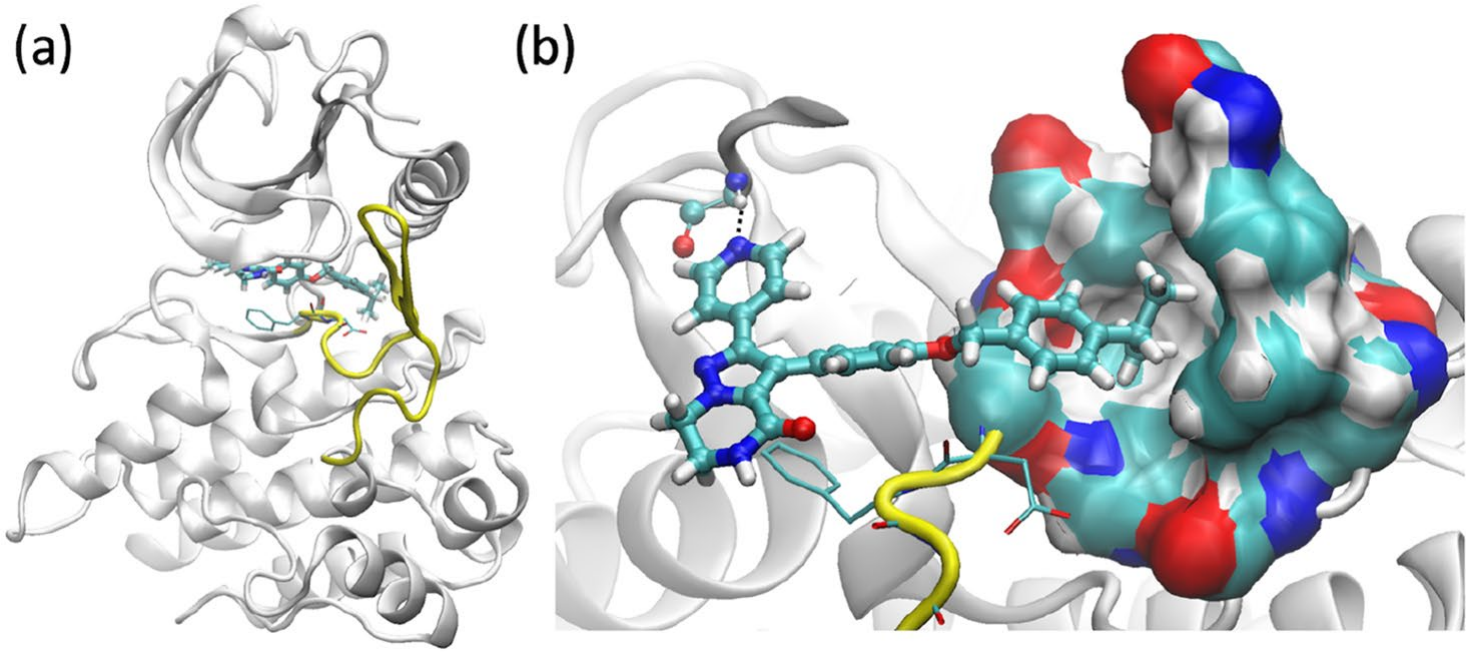
Structures of ROS1 complexed with compound L2: (**a**) overall structure and (**b**) the binding site, showing the hydrophobic binding pocket and a hydrogen bond (dashed line) of the compound with one hinge residue. The compound is shown in ball-and-stick representation. The activation loop (yellow), beginning with a conserved DFG motif (thin sticks), adopts an inactive DFG-out conformation.

Chain A of one of the Janssen structures has been used for the current study, in which ROS1 adopts the DFG-out conformation (Fig. 2). The missing residues in the A-loop are constructed by ModLoop^25^. All water molecules in the pdb file are retained. There are three phosphorylated tyrosine residues in the X-ray structures; they are phosphorylated as well in the simulations. The compounds were docked with the protein using the OpenEye Posit method^26^, drawing on information from the bound ligands to improve pose prediction. All the compounds share the same binding pose at the ROS1 binding site, forming one hydrogen bond with the hinge residue (Fig. 2). The docked structures for all of the compounds have the scaffold well aligned with that in the X-ray structure. They are used as the initial structures for ESMACS simulations. For each of the hybrid compounds in the TIES simulations, the docked position for one of the two compounds is used while additional atoms from another compound are placed accordingly by TIES20^27^ (https://ccs-ties.org) to retain their original conformation. No obvious steric clashes are detected between the hybrid compounds and the protein in any of the initial TIES models.

Preparation and setup of the simulations were implemented using the BAC (binding affinity calculator)^23^, including parametrization of the compounds, solvation of the complexes, electrostatic neutralization of the systems by adding counterions and generation of configurations files for the simulations. Protein parameters were taken from the standard Amber force field for bioorganic systems (ff14SB)^28^. Ligand parameterizations were produced using the general Amber force field 2 (GAFF2). The partial charges of the compounds were generated using AM1-BCC method which has been suggested to be preferable compared with the RESP method^22^.

All ligand–protein complexes were solvated in orthorhombic water boxes with a minimum extension from the protein of 14 Å. The protonation states of the residues were assigned using the “reduce” module of AmberTools^28^. Counterions were added to electrostatically neutralize the systems.

### ESMACS

We use the ESMACS^14^ protocol for the free energy calculations. We also used TIES^15^ for the prediction of relative free energies, described later. The protocols use ensembles of replicas to obtain reproducible binding affinity estimates with robust uncertainty estimates. ESMACS is based on MMPBSA calculations but incorporates a variety of sampling approaches and entropic calculations^29, 30^. Here conformations of the complex, receptor and compound are all extracted from simulation of the complex, a protocol termed as 1traj^31^. This is the commonly used approach for the end-point free energy methods. It achieves good convergence because of the cancellation between the noisy terms of the internal energies of the ligand, receptor and complex. Upon binding, however, conformational changes occur for both protein and ligands, which associate with adaptation free energies^14^. They are the energy differences between the free state and the bound state. Studies have shown that the inclusion of adaptation energies clearly improves the predictions of binding free energy ranking for some molecular systems^14, 29, 32^, while the situation may be more complicated for others^30, 31^. The entropic contributions are commonly calculated by normal mode analysis. The computational cost and numerical instability of normal mode approach^33^, however, have motivated alternatives for the entropy calculations, including interaction entropy^34^, weighted solvent accessible surface area^35^, and external entropy correction^33^. Although the inclusion of entropic contribution can bring the estimated free energies closer to more physically realistic values, it fails to improve correlations in most cases^30, 31, 36, 37^. For a rational drug development project, the correct ranking of binding affinities is more important for the selection of compounds for further investigation. In the current study, the entropic contribution is omitted in the ESMACS free energy calculations. The binding free energies of multiple compounds are investigated to the same protein receptor. The adaptation energy can be calculated in relative terms using the average energy of the protein^29^, which is then incorporated into 1traj, a protocol designated as 1traj-ar^31^.

Ensembles of NAMD^38^ simulations with 25 replicas were performed, with different initial velocities drawn independently from a Maxwell–Boltzmann distribution. A series of equilibration runs, totalling 2 ns, were conducted, with the restraints on heavy atoms gradually removed. Finally, 4 ns production simulations were run for each replica for all ESMACS and TIES simulations. For the ESMACS free energy calculations, the conformations of the protein and the ligands were extracted from the complex simulations.

### TIES

We use TIES^15^ to predict free energy differences for two groups of compound pairs, using compounds L30 and L2 as the reference structures. The two compounds differ by an isopropyl group which extends into a hydrophobic binding pocket (Fig. 2b). TIES20^27^ is used to identify the maximum common substructure and to generate the topology and coordinate files for the hybrid compounds. A total of 103 compound pairs have been constructed using the two reference compounds. The compound pairs are chosen so that the atoms in the reference compounds are largely in the common region, with no more than 5 atoms in the alchemical region from each of the compounds. The standard TIES protocol^15, 27^ is used, in which an ensemble of 5 replicas is used for each pair of the compounds. The TIES toolkit (https://ccs-ties.org/) has been used to automate all of these steps for this study.

While the first group mainly focuses on the differences at the hydrophobic site, the compounds in the second group share the same isopropyl group at the hydrophobic site but differ at other functional site(s) (Fig. 1). Compound L2 is in both of the groups, and is one of the best binders in the first group. The grouping indeed represents the common lead optimisation steps: optimising one functional position of the lead to find more promising compounds, and then adding/changing functional groups at other positions.

The majority of TIES simulations—74 out of 103—are performed with NAMD. The remaining 29 pairs are studied with OpenMM^39^. The two MD engines were used to take advantage of the architectures of different supercomputers. We use NAMD on CPU-based ARCHER2 (https://www.archer2.ac.uk/) and SuperMUC-NG (https://doku.lrz.de/display/PUBLIC/SuperMUC-NG), and use OpenMM on the GPU partition of Summit (https://www.olcf.ornl.gov/summit/). Our previous studies have shown that, for the same molecular systems, consistent free energies are obtained not only from different supercomputers using the same MD engine—NAMD in this case^32^, but also from different MD engines on different supercomputers^21, 40^—NAMD^38^ running on CPUs and OpenMM^39^ or pmemdGTI^41^ running on GPUs.

## Results

### ESMACS results

**Figure 3:**
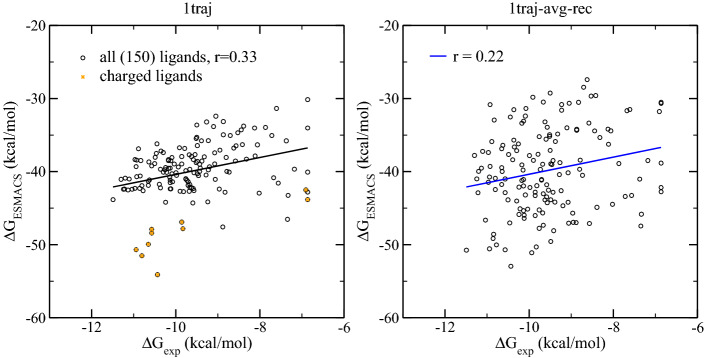
Comparison of calculated binding free energies and experimental binding affinity data from 1traj and 1traj-ar ESMACS approach. The charged compounds are shown in orange.

A moderate correlation, r = 0.33, is obtained for the entire set of the compounds (Fig. 3). The predicted binding free energies appear to be more negative for the charged compounds. These compounds have no obvious differences with the electrostatically neutral ones in their structural and dynamic behaviours. The inclusion of receptor adaptation energy in the 1traj-ar approach causes the calculated binding free energies to be scattered more widely, with a reduced correlation coefficient (r = 0.22).

The scaffold is aligned well for all the compounds in their initial structures; it has the same pose as shown in Fig. 2. For the compounds with similar experimental binding affinities, the free energies of the charged ones are more favourably predicted than the electrostatically neutral ones (Fig. 3). A good correlation is obtained for the charged compounds, with a correlation coefficient of 0.84. This is largely due to the correct predictions of the ranking for the two compounds with less favourable binding affinities (Fig. 3). The correlation coefficient exhibits no improvements when the charged compounds are excluded, having the same correlation coefficient of 0.33 but a reduced covariance from 1.37 to 1.11 kcal/mol. The weak correlation is due to the wide spread of the predictions for the compounds with less favourable binding affinities. More negative predictions are made for some of these compounds (Fig. 3), which are caused by more negative van der Waals contributions. It should be noted that, because of the exclusion of entropic contributions, the predicted binding free energies are much more negative than the experimental measurements. The inclusion of entropy terms typically reduces errors but does not improve the correlations, as noted in previous studies^30, 31, 36, 37^.

**Figure 4:**
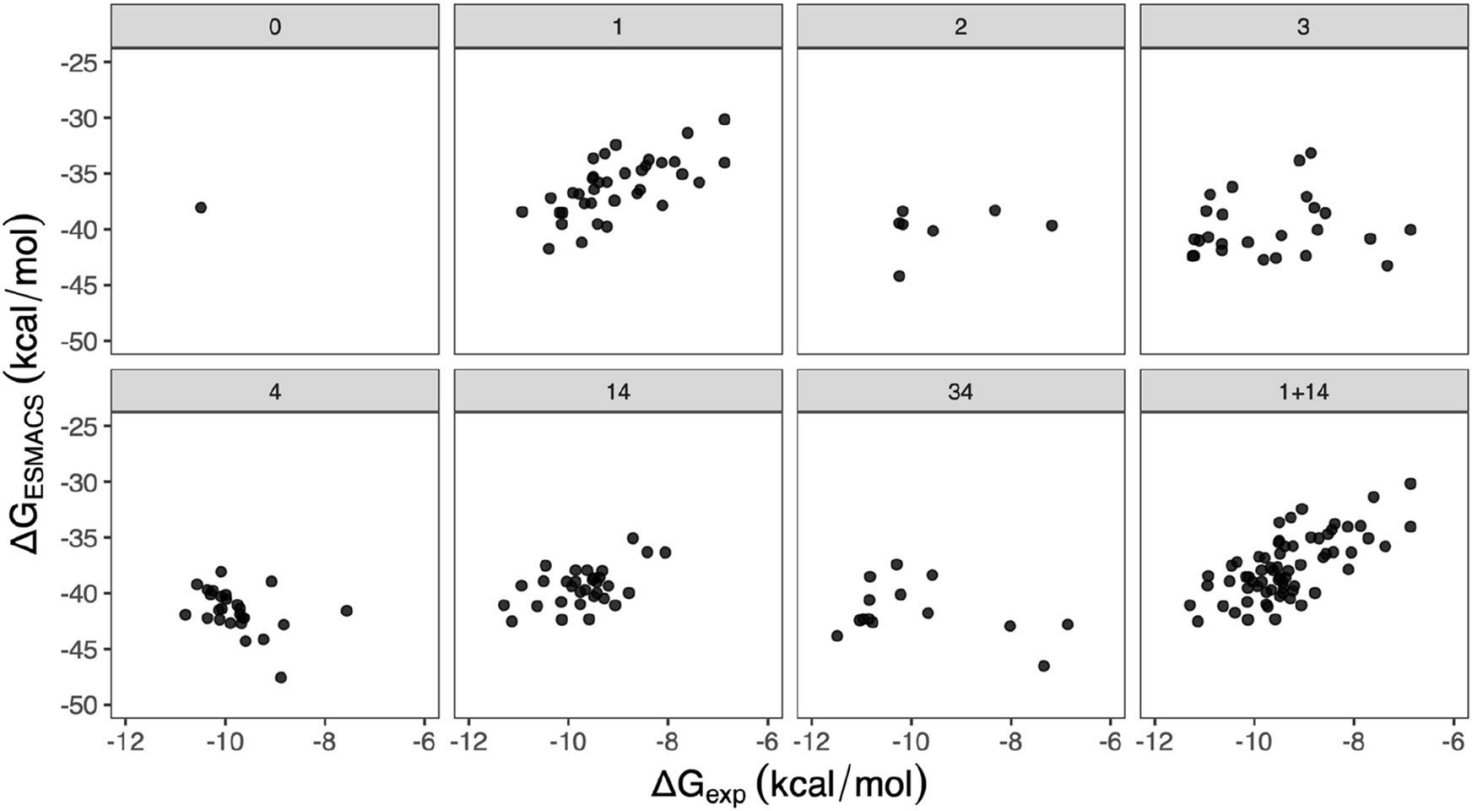
Free energy differences from ESMACS for the electrostatically neutral compounds, grouped into subsets of clusters. In each subset structural modification to L30 (denoted as 0) is restricted to specific regions, with increasing numbers of regions being included. The sets are: (1) ether variations, (2) pyridyl variation, (3) central aryl variation, (4) lactam substituent variation. The numbers (1–4) and their combinations also refer to the different data sets displayed in the panels.

Based on the positions and the chemical properties of the modifications made to the scaffold (Fig. 1, Table 1), the compounds are clustered into different groups. The grouping is based on the following categories: (1) ether variation at position hy in Fig. 1, (2) pyridyl variation at position hg, (3) central aryl variation at position b, (4) lactam substituent variation at position s. About one third of the compounds have modifications at more than 1 positions. This results in a division of the dataset into 7 subgroups: 0, 1, 2, 3, 4, 14 and 34. Subgroup 0 consists of the sole compound L30 (Fig. 1b), subgroups 1–4 have compounds with modifications (1)–(4), respectively, subgroup 34 has compounds with modifications (3) and (4), and subgroup 14 has compounds with modifications (1) and (4), and additional modification (3) for 4 of the compounds. These 4 compounds are grouped into 14 as the modification (3) is minor, with only one hydrogen atom being replaced by a fluorine atom, which should not affect the ligand–protein interactions significantly. At position s, a diverse modification is introduced in subgroup 4, with different sizes, up to 10 heavy atoms, and different chemical properties; a limited modification is applied at this position in subgroups 14 and 34, with no more than 5 heavy atoms added there. The subgroup within category 1 shows a strong correlation (r = 0.66), while additional modification within category 4 (subgroup 14) weakens the correlation slightly (r = 0.52) (Fig. 4). The combination of the two subgroups, containing about half of the neutral compounds, yields a similar correlation to that for subgroup 1. The compounds in these two subgroups have functional groups modified at positions s and hy. The position is located at the solvent accessible region of the binding pocket, while the position hy is accommodated in a relatively spacious hydrophobic region. No significant conformational changes are needed from protein to accommodate the functional groups for compounds in subgroups 1 and 14. The 1traj protocol and the simulation length used here work well for such cases. By contrast, the modifications at position hg (subgroup 2) are likely to affect the hydrogen bond of the ligands with the hinge residue, the modifications of the aryl group at position b (subgroup 3) affect the interactions of the compounds with the buried region where the gatekeeper residue locates, and large modifications at position s (subgroup 4) disturb the solvent exposure region. The changes at these positions influence the conformation of the binding pocket, especially the adenine and buried regions. The compounds in these subgroups show weak or very weak correlations/anticorrelations (Fig. 4, Table 1), indicating that long simulations may be needed to fully sample the conformational changes. The performance of the predictions for different subgroups is in agreement with the criteria we have proposed^42^ for the quality of predictions, including how well a compound fits into the binding pocket and how many rotamers a compound may manifest. While the inclusion of adaptation energies does not improve the overall correlation (Fig. 3b), it removes the anticorrelations for the subgroups 4 and 34, with correlation coefficients modified from −0.39 and −0.37 to −0.04 and 0.09, respectively.

**Table 1:** Correlations of calculated and experimental binding free energies for the subgroups of the compounds.

Subgroup^a^	0	1	2	3	4	14	34	1 + 14
No. of comp	1	36	7	25	26	31	14	67
Pearson r	–	0.66	0.28	0.10	−0.39	0.52	−0.37	0.68
Spearman ρ	–	0.66	0.18	0.21	−0.49	0.37	−0.06	0.61
Kendall τ	–	0.48	0.15	0.14	−0.40	0.24	0.03	0.43

^a^The subgroups are defined using single categories (1–4) or combinations of these categories. 14, for example, is a subgroup of compounds with modifications of (1) ether variation and (4) lactam substituent variation. “1 + 14” is a subset of compounds from subgroups 1 and 14. L30 is labelled as the only member in subgroup 0. Only electrostatically neutral compounds are included.

### TIES results

The TIES study was performed for a total of 103 compounds pairs, of which 29 are simulated by OpenMM and 74 by NAMD. Our previous studies have shown that consistent results are generated from different MD engines^21, 40^ or on different computational platforms^32^, provided that the same force field is used and the ensemble approach is employed. Here we evaluate the performance of TIES protocols in reproducing the experimental data for the dataset.

#### Comparison of predictions with experimental data

**Figure 5:**
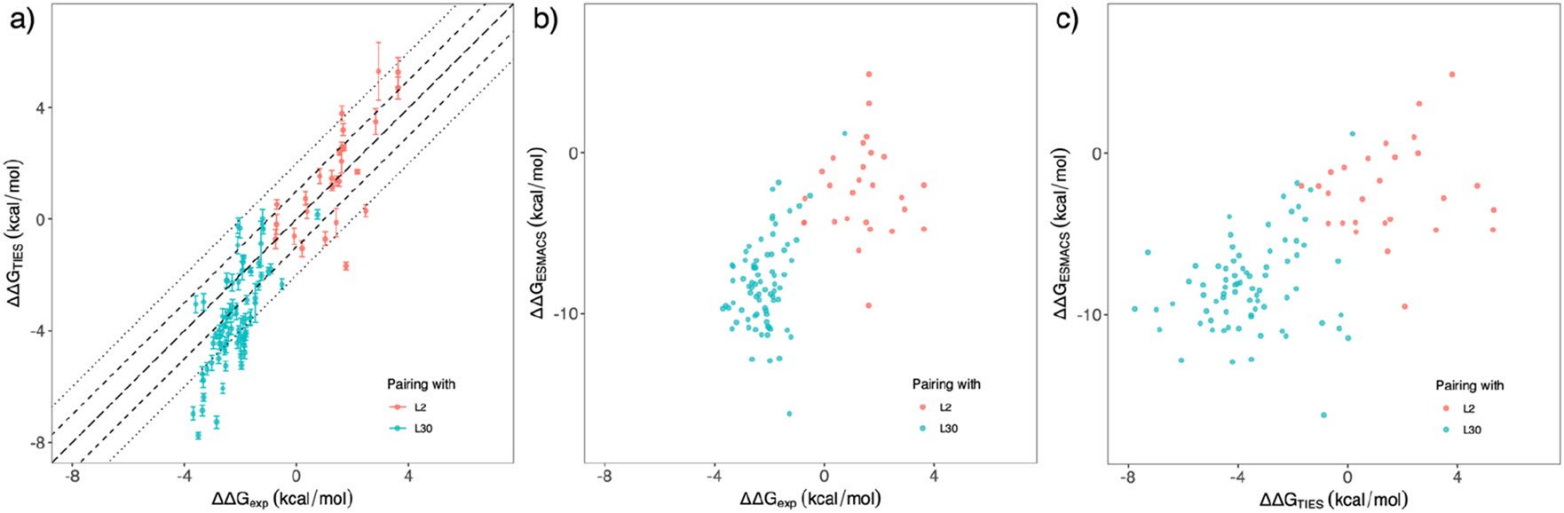
Comparison of relative binding affinities from (**a**) TIES and experiment, (**b**) ESMACS and experiment, and (**c**) TIES and ESMACS for the compound pairs studied in TIES. In subfigure (**a**), the long dashed, short dashed and dotted lines are y = x, y = x ± 1 and y = x ± 2. An excellent correlation is obtained between the TIES calculations and the experimental measurement, with a correlation coefficient of 0.90. Strong correlations are also obtained between ESMACS and experiment, and between TIES and ESMACS, with correlation coefficients of 0.67 and 0.62, respectively.

A very good correlation, with a correlation coefficient of 0.90, is obtained between the calculated free energy differences and the experimental data (Fig. 5a). In the subgroup with compound L30 as the reference, most of the predicted binding free energy differences are negative, indicating that the modifications to the compound are likely to improve the binding potencies (ΔΔG < 0). The calculations correctly predict compound L2 (Fig. 1) as one of the best binders. As L2 already has a very favourable binding free energy (−10.49 kcal/mol), it is not surprising that most of the modifications from it deteriorate the binding affinities (ΔΔG > 0). For all of the 103 compound pairs, 94% of the TIES predictions agree directionally with the experimental observations, that is, the calculated binding free energy differences have the same sign as those from experimental measurements.

The relative binding free energy differences from TIES agree well with those from ESMACS (Fig. 5c), with a correlation coefficient of 0.62. The predicted ΔΔG values for the two subgroups, paired with L30 and L2, are clearly distinct from both of the protocols. The predicted ΔΔG results from ESMACS also have a good correlation with the experimental data, with a correlation coefficient of 0.67 (Fig. 5b). We should again point out that because of the nature of the approximations used, such as use of an implicit solvent model and the neglect of the conformational entropy contributions, the ESMACS approach does not provide accurate absolute (Fig. 3) or relative (Fig. 5b) binding free energies. However, it often yields precise binding affinity rankings. As an alchemical approach, TIES is in principle both accurate and precise in its domain of applicability. The calculated binding free energies are therefore directly comparable with those from experimental measurements, as evidenced by the same scales of the axes in Fig. 5a.

It should be noted, however, that an apparent overestimation is observed for some of the compound pairs involving L30, making the predicted free energy differences larger than those from experiments. An MSE of −1.36 kcal/mol is obtained for the L30 subgroup, compared with an MSE of 0.16 kcal/mol for the subgroup with compound L2 as the reference. The apparent overestimations are investigated further in the following sections.

#### Overestimation from closed cycle mutations

**Figure 6:**
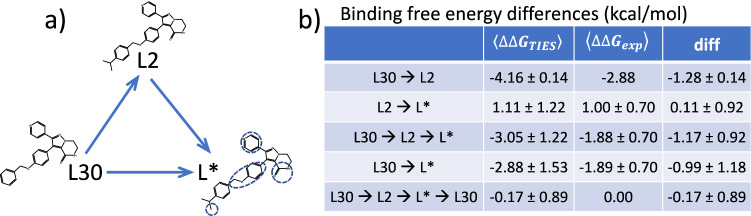
Binding free energy differences in the closed cycle. L* represents 17 compounds which have differences at one or more positions (blue cycles) to L2, and have been paired with both L30 and L2. The errors for L30 → L2 are derived from the bootstrapped standard error from the ensemble simulations. The errors in all other energy terms are the standard deviations of sampling distributions from the 17 compounds. The experimental measurements are reported as single values with no uncertainties provided.

There are 17 compounds which are paired with both L30 and L2 (Fig. 6). This makes it possible to check the performance of the cycle closure method. For the transformations starting from L2 (L2 → L* in Fig. 6a), the average free energy change from TIES calculations agrees well with the experimental data (Fig. 6b). The transformation L30 → L2, however, overestimates the difference by 1.28 kcal/mol. The two-step transformation for all 17 compounds (L30 → L2 → L*) generates an averaged overestimation of 1.17 kcal/mol, similar to that observed in L30 → L2 transformation. Compounds L* and L2 (Fig. 6) have the same/similar alkyl isopropyl substituent at the hydrophobic position (Fig. 1), while L30 does not; transformations L30 → L* therefore share the same change as that in L30 → L2 transformation. There is a similar overestimation in L30 → L* (−0.99 kcal/mol) as that in L30 → L2, which is likely to be rooted in the same alchemical change of growing an alkyl group from L30.

The legs L30 → L2, L2 → L* and L* → L30 generate an average difference of 0.17 kcal/mol within the closed cycle (L30 → L2 → L* → L30, Fig. 6) between the calculations and the experiments. As we have stated previously^40^, a hysteresis value of 0 is a necessary but not sufficient condition for convergence of predictions. An overall hysteresis near 0 for a closed cycle is not necessarily an indication of convergence for each leg; it may be a result of cancellation from individual legs. It is understandable that extending an alkyl group into a hydrophobic binding pocket (Fig. 2b) makes the binding more favourable, about which TIES and experiment agree with each other directionally. It is likely that the force field and/or the sampling contribute to the differences between calculations and experimental measurements. All simulations were initiated from the same X-ray structure in which an isopropyl group is present in the bound ligand. The removal of the group (Fig. 1) would be expected to induce some conformational collapse or solvation of the hydrophobic subpocket (Fig. 2). The subpocket is formed by a few hydrophobic residues including Leu2000, Phe2004, Ile2009, Leu2070, Phe2075, His2077 and Ile2100. The root-mean-square deviations of these residues from the X-ray structure show no overall differences for the simulations of L2 and L30. Therefore, despite the variability in the ligand structure, no significant changes are observed in the shape and size of the subpocket from the simulations of the two ligands. This indicates that the current sampling regimen does not permit protein conformational adaptations for ligands without the isopropyl group in the ESMACS and TIES simulations. This could explain the predicted less favourable binding and hence larger differences in the binding free energies (Figs. 5a, 6) between the ligands with and without the isopropyl group. It is unlikely though that ROS1 needs to adopt a DFG-in conformation to accommodate the compounds containing the –CH group, as the DFG motif directly interacts with the scaffold of the compounds and retains them in the same binding mode (Fig. 2).

#### Ligand pairs sharing the common change

**Figure 7:**
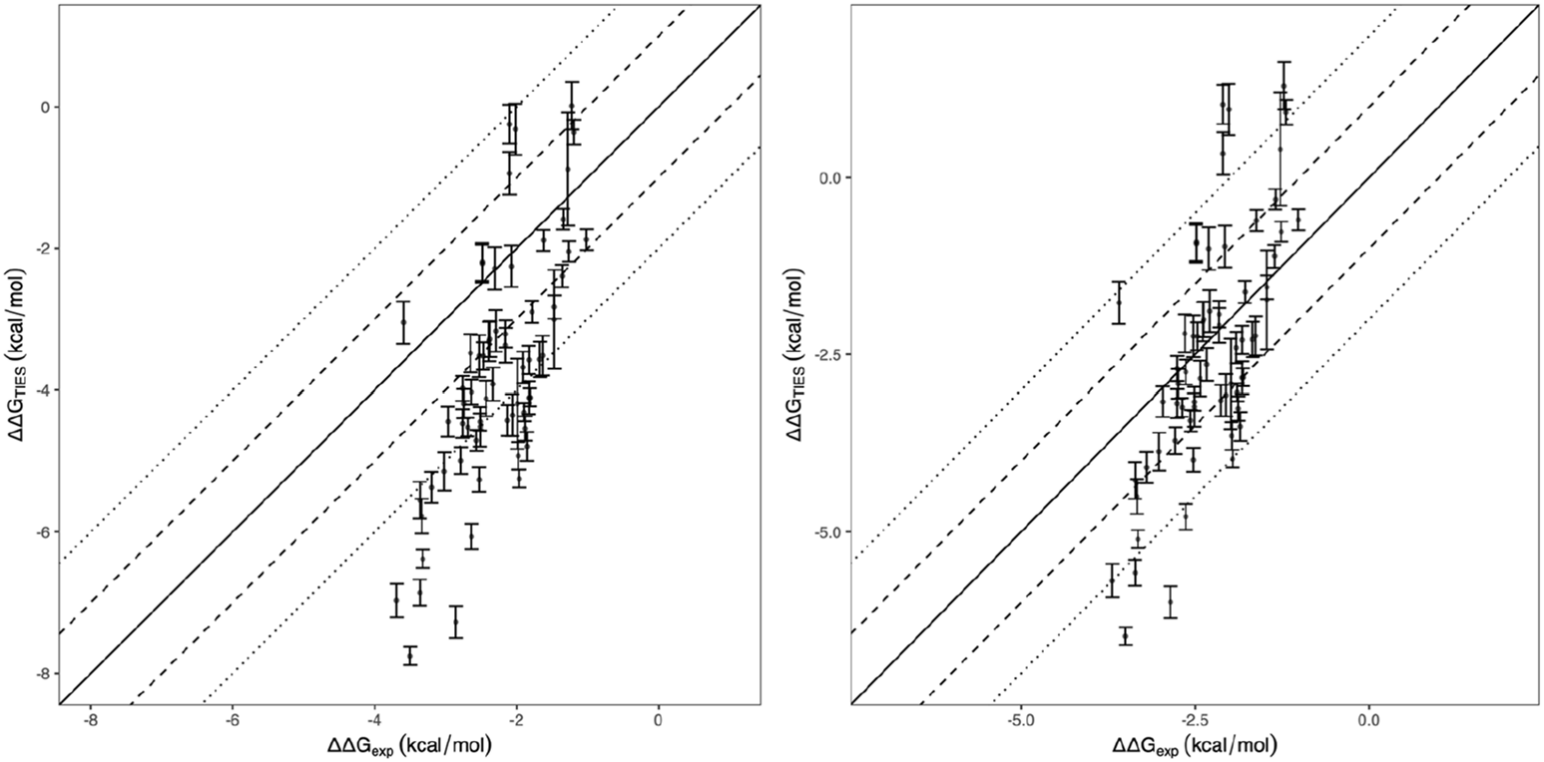
Original and “corrected” binding free energies from TIES calculations, compared with the experimental data. The correction is made by assuming a systematic overestimation is introduced by –CH to –C–CH–(CH_3_)_2_ transformation which is involved in all of these pairs.

50 pairs have been identified which involve a common variation between the two compounds in the pairs, changing from a –CH group to –C–CH–(CH_3_)_2_ at the hydrophobic site. This is the same change as that between L30 to L2 (Fig. 1). Simulations for the pair get an overestimation of 1.28 kcal/mol for the binding free energy difference: −4.16 kcal/mol from TIES vs −2.88 kcal/mol from experiment. For these 50 pairs, an averaged binding free energy difference of −3.82 kcal/mol is obtained from TIES calculations, while an average of −2.42 kcal/mol from experimental data. A recent study has proposed a combination of machine learning (ML) and alchemical free energy calculation to improve the accuracy of free energy predictions^43^. The ML method is used to learn the differences between the calculations and the experimental data and derives a correct term which brings the two closer. Such an approach may identify and subsequently correct the discrepancies as observed here. It should be noted that the ML approach used in the study^43^ only learns the differences between the calculations and experiments, with no attempt to identify the source of the discrepancies. Studies have shown that a large set of typical chemical modifications lead to a nearly normal distribution of binding energy differences^44^. For individual modifications, however, a wide and non-Gaussian distribution has been observed for experimental binding free energies from a large number of independent measurements^42^. Such non-normality has also been reported in numerous ensemble simulations when large sample sizes are used^20, 22, 27^. These observations indicate that the assumption of Gaussian statistics may not be justified^20,42^. If, on average, these 50 compound pairs have the same discrepancy as that in L30 → L2 transformation, the systematic differences might be collectively “corrected”. If we apply an offset and shift up all of the TIES results by 1.28 kcal/mol, the calculations agree well with the experimental data, with an MSE reducing from −1.40 to −0.12 kcal/mol (Fig. 7). As stated above, this offset could be associated with incomplete sampling of the protein conformational movement that is required to fully accommodate the change from a –C–CH–(CH_3_)_2_ group, which is present in the initial X-ray structures, to a –CH group in the region of the A-loop.

## Conclusion

Using the TIES and ESMACS protocols, we have computed the binding free energies of a series of ligands interacting with ROS1 kinase. Good correlations are obtained from our ESMACS study for subsets of the compounds. An excellent statistical ranking is generated from the TIES study, with a correlation of 0.90 between the predictions and the experimental measurements. Moreover, TIES and ESMACS agree with one another in the prediction of free energy differences.

An unexplained overestimation is observed in the TIES study for one subset of compound pairs which share a common difference between the pairs. For the transformations to which cycle closure methods can be applied for the error estimation, a cancellation has been observed as the errors are similar for specific legs within the cycle. This results in an overall hysteresis near 0 for a closed cycle, but significant errors remain for individual legs. While continuing improvements in the force fields and the application of statistically robust ensemble methods are essential to generate increased accuracy for the physics-based alchemical approaches, machine learning methods may help to identify discrepancies between the predictions and experimental data and potentially reduce those differences, providing sufficient quantities of reliable training data are available that incorporate information on the way such data are distributed. Despite the existence of this systematic discrepancy, an excellent correlation is obtained in the free energy differences between the calculations and the experiments, showing once again that the TIES approach can be used in prospective drug discovery projects to predict the changes of binding free energies in the lead optimisation process^29, 32, 37^.

## Data Availability

The structures for the ROS1 protein and the compounds, and Amber format topologies and PDB-format coordinates for the compound pairs, are freely available at http://doi.org/10.23728/b2share.89da477f828048dd833272b51360d13a.
